# Revealing ferroelectric switching character using deep recurrent neural networks

**DOI:** 10.1038/s41467-019-12750-0

**Published:** 2019-10-22

**Authors:** Joshua C. Agar, Brett Naul, Shishir Pandya, Stefan van der Walt, Joshua Maher, Yao Ren, Long-Qing Chen, Sergei V. Kalinin, Rama K. Vasudevan, Ye Cao, Joshua S. Bloom, Lane W. Martin

**Affiliations:** 1 Department of Materials Science and Engineering, University of California, Berkeley, Berkeley, CA 94720 USA; 2 Materials Sciences Division, Lawrence Berkeley National Laboratory, Berkeley, CA 94720 USA; 3 Department of Materials Science and Engineering, Lehigh University, Bethlehem, PA 18015 USA; 4 Department of Astronomy, University of California, Berkeley, Berkeley, CA 94720 USA; 5 Berkeley Institute of Data Science, University of California, Berkeley, Berkeley, CA 94720 USA; 6 Department of Materials Science and Engineering, The University of Texas at Arlington, Arlington, TX 76019 USA; 7 Department of Materials Science and Engineering, Pennsylvania State University, University Park, PA 16802-5006 USA; 8 Center for Nanophase Materials Sciences, Oak Ridge National Laboratory, Oak Ridge, TN 37830 USA

**Keywords:** Ferroelectrics and multiferroics, Computational science, Surfaces, interfaces and thin films

## Abstract

The ability to manipulate domains underpins function in applications of ferroelectrics. While there have been demonstrations of controlled nanoscale manipulation of domain structures to drive emergent properties, such approaches lack an internal feedback loop required for automatic manipulation. Here, using a deep sequence-to-sequence autoencoder we automate the extraction of latent features of nanoscale ferroelectric switching from piezoresponse force spectroscopy of tensile-strained PbZr_0.2_Ti_0.8_O_3_ with a hierarchical domain structure. We identify characteristic behavior in the piezoresponse and cantilever resonance hysteresis loops, which allows for the classification and quantification of nanoscale-switching mechanisms. Specifically, we identify elastic hardening events which are associated with the nucleation and growth of charged domain walls. This work demonstrates the efficacy of unsupervised neural networks in learning features of a material’s physical response from nanoscale multichannel hyperspectral imagery and provides new capabilities in leveraging in operando spectroscopies that could enable the automated manipulation of nanoscale structures in materials.

## Abstract (web summary)

The scale and dimensionality of imaging data means information is commonly overlooked. Here, using recurrent neural networks we understand temporal dependencies in hyperspectral imagery, enabling the observation of differences in ferroelectric switching mechanisms in PbZr_0.2_Ti_0.8_O_3_ thin films due to formation of charged domain walls.

## Introduction

The ability to create and manipulate domain structures in ferroelectrics allows for the control of the phase and polarization orientation and the local and macroscale susceptibilities (e.g., electrical, thermal, mechanical, optical), thus providing a foundation for next-generation devices^1–3^. Early efforts in this regard have focused on deterministically creating desired domain structures. In, for example, tetragonal PbZr_1−*x*_Ti_*x*_O_3_, controlling the elastic boundary conditions has provided access to domains spanning simple monodomain to complex hierarchical domain structures^4–6^. As the field has advanced, ingenious routes, including compositional gradients^7,8^, superlattice structures^9–11^, orientation control^12,13^, and engineered octahedral rotations^14^, have been leveraged to control domain structures.

The majority of this work, however, has focused on the static creation of desired domain structures^6,15–18^ or functional domain walls,^19,20^ and thus lacks an internal self-regulating feedback loop required for automatic operation in functional devices. To deterministically manipulate ferroelectric domain structures requires the ability to measure while in operation (i.e., in operando) and automatically identify a number of features (e.g., polarization orientation, switching pathways, domain-wall geometry). Developments in multimodal spectroscopy now allow the acquisition of data at both the appropriate time- and length scales required to glean such information from ferroelectric materials using techniques such as transmission electron microscopy^21,22^, scanning-probe microscopy^23,24^, diffraction studies^25,26^, etc.^27^ The challenge, however, is that downstream analytical approaches which project data into a human-interpretable form remain underdeveloped and ill equipped for the complexity and magnitude of the data that can now be readily produced. In turn, despite the extensive amount of expensive experiments conducted, only an infinitesimally small fraction of the data collected is translated into knowledge.

Solving this challenge requires looking beyond the borders of nanoscience to fields such as social analytics^28,29^, natural-language processing^30,31^, and sentiment analysis^32,33^, where computational roadblocks are pervasive. For decades, standard practice was to develop machine-learning algorithms to create mathematical abstractions of the data based on characteristics of preconceived importance. Recently, the availability of massive datasets and specifically designed hardware has enabled features once designed by human experts to be extracted using brute-force computation. These representation learning tools generally rely on building architectures of simple non-linear mathematical functions which are optimized to relate the raw data to some information or label^34–36^. These so-called deep-learning-neural-network-based approaches have set new benchmarks for many common machine-learning tasks including image^37^ and speech recognition^38^, language translation^39^, and identification of human intention^32,33^. While these deep-learning approaches have begun to make meaningful inroads in, for example, genomics^40^, high-energy physics^41^, and astronomy^42^, they have yet to be sufficiently embraced in experimental nanoscience^43–53^.

Here, we develop a sequence-to-sequence neural network to extract inference from band-excitation piezoresponse spectroscopy (BEPS). To test our approach, we conducted BEPS on tensile-strained PbZr_0.2_Ti_0.8_O_3_ thin films wherein strain drives the formation of a hierarchical *c*/a and *a*_1_/*a*_2_ domain structure. We develop and train a deep-learning-neural-network-based sparse autoencoder on piezoresponse hysteresis loops to demonstrate parity with conventional empirical-analysis approaches. We then apply this approach to extract insight from the resonance response which has a form too complex to be properly analyzed using techniques common in experimental materials science. Using the information “learned”, we identify geometrically driven differences in the switching mechanism which are related to charged-domain-wall nucleation and growth during ferroelastic switching. This insight could not have been extracted using machine-learning approaches that have been previously applied to materials spectroscopy and provides unprecedented information about the nature of the specific domain-structure geometries that should be explored to enhance local and macroscale susceptibilities. Furthermore, the ability to automate the extraction of inference regarding ferroelectric-switching mechanisms from multichannel nanoscale spectroscopy provides the first step (i.e., machine-learned discrimination) that could be used to design real-time control systems capable of creation and verification of interconversion of functional domain structures and interfaces. The developed approach is extensible to other forms of multi-dimensional, hyperspectral images (wherein there is a spectra at each pixel) which are commonly acquired in experiments such as time-of-flight secondary-ion mass spectrometry^54,55^, scanning Raman^56^, electron energy loss spectroscopy^57,58^, etc. To promote the utilization of this approach, we provide open access to all data and codes in the form of a Jupyter notebook^59^ (Supplementary Note 1). Ultimately, this work represents an example of how unsupervised deep learning can highlight features relating to ferroelectric physics overlooked by human-designed-machine-learning algorithms, and how such approaches can be adapted to analyze hyperspectral data more broadly.

## Results

### Synthesis of PbZr_0.2_Ti_0.8_O_3_ thin films with hierarchical domain structures

**Fig. 1:**
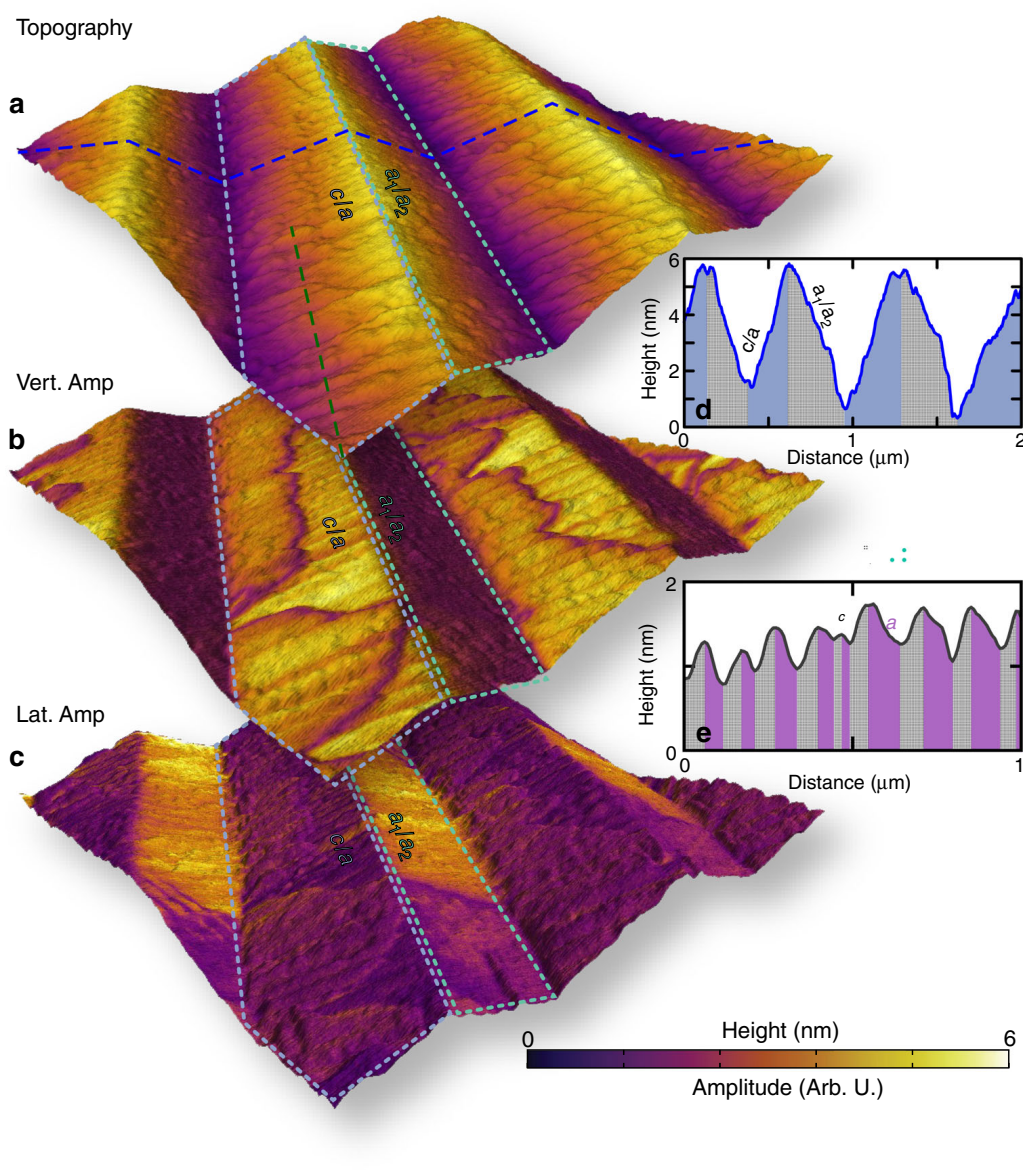
Surface topography of PbZr_0.2_Ti_0.8_O_3_ with hierarchical domain structures. **a** Three-dimensional tapping mode topography superimposed with topographic information presented in nm as indicated. **b**, **c** Three-dimensional tapping mode topography superimposed with the **b** vertical and **c** lateral piezoresponse amplitude. Color presented in arbitrary units defined by the color scale in the inset. Topographic line traces showing the surface topography across the **d** *c*/*a*-*a*_1_/*a*_2_ bands (indicated by the dark-blue-dashed line in **a**) and **e** *c*/a bands (indicated by the dark-green-dashed line in **a**)

We synthesized PbZr_0.2_Ti_0.8_O_3_/Ba_0.5_Sr_0.5_RuO_3_/NdScO_3_ (110) heterostructures using pulsed-laser deposition (Methods). The resulting films have a hierarchical domain structure with a sawtooth topography on two length scales (Fig. 1a–e), as the result of primarily out-of-plane polarized *c*/*a*/*c*/*a* [with enhanced out-of-plane (Fig. 1b) and suppressed in-plane (Fig. 1c) piezoresponse] and fully in-plane polarized *a*_*1*_/*a*_*2*_/*a*_*1*_/*a*_*2*_ [with suppressed out-of-plane and enhanced in-plane piezoresponse] domain bands. This hierarchical domain structure emerges due to the tensile strain which drives the *c*/a and *a*_*1*_/*a*_*2*_ domain variants to be nearly energetically degenerate (Supplementary Note 2)^6^.

### Band-excitation piezoresponse force microscopy

To characterize the nanoscale-switching processes, we conducted BEPS (Methods and Supplementary Note 3, Supplementary Figs. 2 and 3). Briefly, BEPS measures the piezoresponse in remanence (across a band of frequencies near the cantilever resonance) following a perturbation from a bipolar-triangular switching waveform designed to fully switch the material. Following fitting, the raw data from this experiment at every pixel (*x, y* size = 60, 60) measures the amplitude (*A*), phase (*ϕ*), resonance frequency (*ω*), and quality factor (*Q*) of the cantilever resonance, which are qualitative measures of the piezoresponse, polarization direction, stiffness, and dampening, respectively, at various voltages (*V* length *=* 96). At the most basic level, visualization of the switching processes can be achieved by creating movies from the images of the signals throughout the switching process or by plotting the response curves at a specific location or within a predefined area (Supplementary Movie 1). Additionally, it is common to compute at each tip position a piezoresponse loop $\left( {A\cos \phi } \right)$ which can then be fit to a 15-parameter empirical function. While such approaches are capable of visualizing large differences in the piezoresponse they provide only limited information into subtle differences of the response, which contains important insight (Supplementary Note 4, Supplementary Fig. 4). While in the raw form the data might occupy an *N*-dimensional space, the information of physical significance lies on a data manifold with a much lower dimensionality; however, we have no means to predict the manifold.

Recognizing these limitations, statistical approaches of machine learning have been applied to predict the data manifold, thus allowing more insight to be extracted from BEPS data. To demonstrate the necessity of the proposed deep-learning approach, we have conducted careful analysis using linear decomposition algorithms, including principal-component analysis and non-negative-matrix factorization, and a variety of clustering algorithms (Supplementary Note 5–7, Supplementary Figs. 5–7). All told, while these methods are able to identify the most significant differences in the response, they have minimal sensitivity to quantify subtle, yet physically significant, features. This is at least in part due to the decoupling of the voltage (i.e., the time or temporal) dependence of the spectra which are viewed as independent samples—meaning that these algorithms exclude the history of when each data point was collected.

### Inference from long–short-term memory recurrent autoencoder

**Fig. 2:**
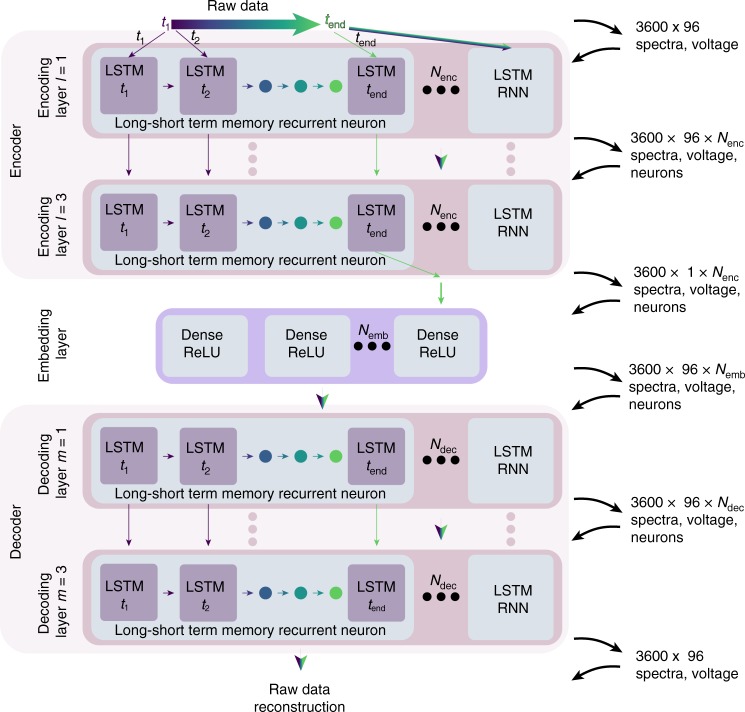
Drawing of sparse long–short-term memory autoencoder. Diagram shows the three components of the neural network the encoder, embedding layer, and decoder. Within each of these sections the dimensionality and size of the input and outputs are indicated on the right. Diagram shows how temporal data (represented as linear color-changing arrows) is consider through the inclusion of recurrent long–short-term memory neurons. Solid colored arrows indicate just a single time step is passed, where arrows with gradients imply the passing of temporally dependent vectors. In the figure *l* is the number of encoding layers, *m* is the number of decoding layers, *N*_enc_ is the number of neurons in the encoding layer, *N*_emb_ is the number of neurons in the embedding layer, and *N*_dec_ is the number of neurons in the decoding layer

Thus, what is required is to develop an approach that considers the temporal dependence inherent in the data. To do this, we developed a sequence-to-sequence deep learning neural network based on a long–short-term memory (LSTM) recurrent neural network (RNN) autoencoder (henceforth called the autoencoder) which acts as a feature extractor to derive inference from BEPS spectra. LSTM neurons (described in Supplementary Note 8, Supplementary Fig. 8) were chosen due to their success in natural language processing (which is analogous in data structure to spectra) wherein order of words (measurements) is important^60^. The autoencoder architecture consists of an encoder, which takes as an input a spectra and outputs a feature vector, and a decoder, which takes this feature vector and returns the input spectra (Fig. 2). By minimizing the mean-squared-reconstruction error of the input spectra the autoencoder “learns” a universal identity function. While building an arbitrarily complex identity function (where the large model capacity assures overfitting) is a fruitless task, building an identity function whose capacity is limited or strongly regularized can produce a generalizable (i.e., with a limited number of characteristic variables) function capable of broader inference.

More explicitly, the first encoding layer accepts a time series as an input which, in this case, is the response from the 96 sequential voltage steps. Each of the encoding layers is composed of LSTM neurons (*N*_enc_ = 256)^61^ with an internal cell structure that enables the retention of long- and short-term temporal dependencies. During training, each layer outputs an abstract representation of the data for each input spectra. To build more descriptive data abstractions, it is typical to stack encoding layers (*l* *=* 3) forming so-called deep neural networks. To minimize overfitting or memorization, dropout (d = 20%)^62^ (which minimizes co-adaptation by randomly removing some of the network connections) was applied. The output from the encoder is then passed to an embedding layer consisting of dense neurons (*N*_emb_ = 16)^63^. This layer creates a low-dimensional representation of different characteristic responses. To make this representation more interpretable, it is beneficial to impose sparsity [i.e., minimize the number of non-zero activations (outputs)]. To do this, two synergistic approaches were applied: First, we restrict the outputs to non-negative values by selecting a rectified-linear activation function (ReLu) ($f\left( x \right) = \max (0,x)$). Secondly, we add strong *l*_1_ regularization which adds an additional contribution to the loss function proportional to the sum of the weights $\left( {\lambda \mathop {\sum }\nolimits_i \left| {W_i} \right|} \right)$, thus only those activations which significantly improve the model’s accuracy are non-zero (this can be visualized in Supplementary Movies 2 and 3). The feature vector is then passed to the decoder which is structured in an identical fashion to the encoder [decoding layers (*m* *=* 3), each with 128 LSTM neurons (*N*_dec_ = 128)]. The decoder takes the feature vector and transforms it back into the original spectra, such that the network can be optimized to minimize the loss function composed of the mean-squared error and the *l*_1_ regularization [$( {{\mathrm{loss}} = \frac{1}{n}\mathop {\sum }\nolimits_{i = 1}^n ( {Y_i - \widehat {Y_i}} )^2 + \lambda \mathop {\sum }\nolimits_i | {W_i} |} )$, Supplementary Note 9, Supplementary Fig. 9].

**Fig. 3:**
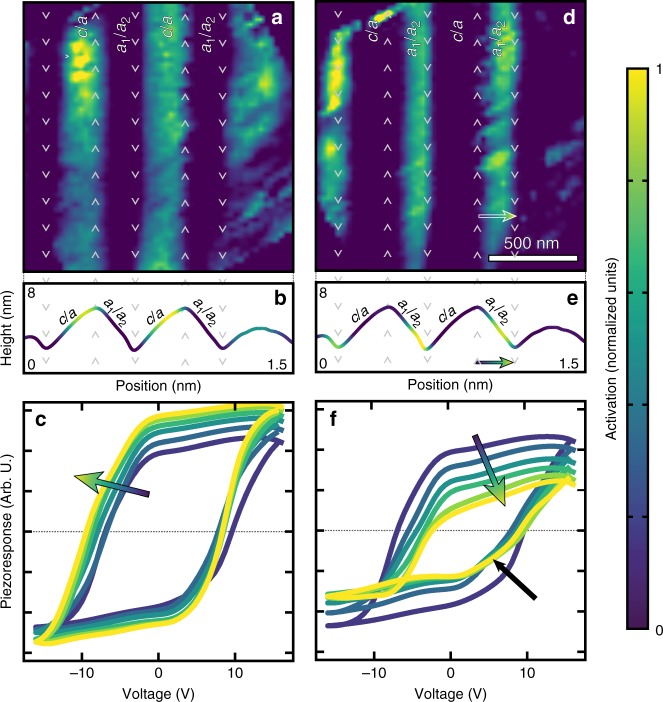
Features learned from low-dimensional layer of the piezoresponse autoencoder. **a**, **d** Feature maps extracted from low-dimensional layer of autoencoder trained on piezoresponse hysteresis loops. Color indicates the magnitude of the latent feature or the activation observed in each spectra at a given pixel position. Activation is mapped in normalized units as shown in colorbar. **b**, **e** Average activation across the domain bands superimposed onto the average topography. **c**, **f** Neural network generated piezoelectric hysteresis loops as the magnitude of the activation is increased. In all figures the color of the curves/images reflect the normalized activation from the low-dimensional representation at that location or from the generated response curve

We begin by training the autoencoder to analyze the piezoresponse hysteresis loops extracted from BEPS (Methods). Since every spectra analyzed by the autoencoder has a known pixel position it is possible to visualize the “learned” information by computing the output of the low-dimensional layer to form a real space feature map. While this layer would permit an independent feature map for each neuron, the addition of sparsity results in most features having a null value. Here, we show two of the most physically meaningful features (Fig. 3a, b), where the intensity of the maps represents the degree of a “learned” characteristic response form. To aid in the visualization of this information we have provided a line trace of the average activation superimposed on the average topography (Fig. 3c, d). The first feature map (Fig. 3a) shows increased activation within the *c*/a bands which is maximized on the peak side of the topographical features (Fig. 3c). To visualize what this activation is encoding, we use the decoder of the autoencoder as a generator, allowing us to manually manipulate the activation to see how this neuron alters the piezoresponse (Supplementary Movie 4). From the generated piezoresponse hysteresis loops, we observe an increase in the magnitude and the squareness of the loops as we increase the activation of this neuron, as would be expected for regions with increasing *c*-like character (Fig. 3e). This suggests that the neuron has “learned” the response associated with *c* domains and, since the map has different magnitudes of response, it provides a way to quantify the *c*-like switching character spatially. In total, this reveals that the autoencoder is capable of deducing physically interpretable inference from the data in an unsupervised fashion. Moving on to the second feature map (Fig. 3b), we notice a gradient in the activation which is maximized within the *a*_*1*_/*a*_*2*_ bands near the valley boundary which decreases in magnitude as we transition towards the peak boundary (Fig. 3d). Visualizing the loops as we increase this activation reveals a decrease in the magnitude and the emergence of an intermediate step in the piezoresponse loop (black arrow, Fig. 3f). This intermediate step is related to a two-step, three-state (*c* → *a* → *c*) ferroelastic switching process^6^.

It is important to reemphasize that this detailed spatial variance was not apparent using conventional machine-learning approaches. All told, this reveals that the autoencoder is capable of “learning” a complex identity function where each neuron controls a physically meaningful characteristic of the piezoresponse. Not only is the autoencoder readily able to differentiate regions of varied response which correlate to different domain structure variants, but it is also able to quantify the relative response character (i.e., how “*x*-like” the response is) which reveals additional complexity. This deviates from traditional approaches which generally provide only qualitative classification of behavior.

**Fig. 4:**
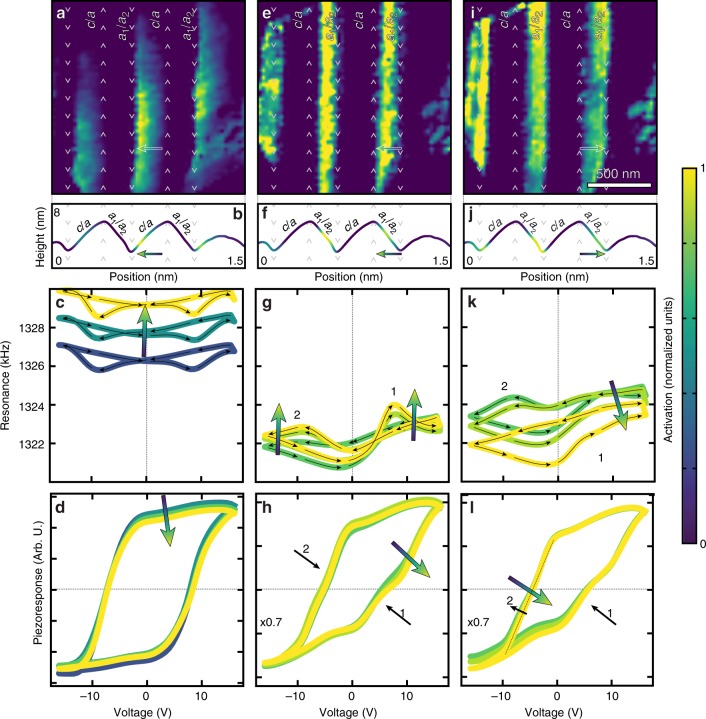
Features learned from low-dimensional layer of the resonance response autoencoder. **a**, **e**, **i** Feature maps extracted from low-dimensional layer of autoencoder trained on the resonance hysteresis loops. Color indicates the magnitude of the latent feature or the activation observed in each spectra at a given pixel position. Activation is mapped in normalized units as shown in colorbar. **b**, **f**, **j** Average activation across the domain bands superimposed onto the average topography. Neural network generated **c**, **g**, **k** resonance hysteresis loops and **d**, **h**, **l** piezoresponse hysteresis loops. In all figures the color of the curves/images reflect the normalized activation from the low-dimensional layer at that location or from the generated response curve. Numbers in figures represent observations of ferroelectric or ferroelastic switching events

Having proven the capabilities of this approach, we applied a similar methodology to interpret the cantilever-sample-contact resonance (henceforth the resonance response) wherein the form of the response has increased complexity which complicates statistical analysis. We show three of the most physically meaningful non-zero components (Fig. 4), obtained following training (Methods). To use the extracted insight to understand the physical mechanisms of response it is important to identify the statistical distribution of the key response characteristics “learned”. The first selected feature map (Fig. 4a) shows increased activation within the *c*/a bands which is maximized near the valley boundary (Fig. 4b). If we again use this autoencoder as a generator (Supplementary Movie 5), we observe that the resonance has a classic butterfly-shaped loop, however, as this activation increases there is a gradual increase in the resonance frequency as we approach the valley boundary (Fig. 4c). If we had followed the traditional approach of merely studying how the piezoresponse hysteresis loops vary as we move from the peak to the valley boundary (Fig.4d), we would have observed essentially no change, thus leaving uncovered new physical effects.

Moving on to the second feature map (Fig. 4e), we observe a gradient in the activation, which is maximized near the middle of the *a*_*1*_/*a*_*2*_ band and tends towards zero at the valley boundary (Fig. 4f). The generated resonance loops reveal a non-traditional shape wherein upon application of bias the material undergoes elastic hardening before eventually softening when switching under both positive and negative bias (marked 1 and 2, respectively, Fig. 4g). This type of resonance behavior during switching has been related to a two-step, three-state (*c* → *a* *→* *c*) ferroelastic switching mechanism^6^. By computing the piezoelectric hysteresis loops, we identify that these loops have an intermediate step which plateaus at near-zero piezoresponse when switching under both positive and negative bias (1 and 2, respectively; Fig. 4h). The generated piezoresponse curves reveal that as we decrease the magnitude of this activation (i.e., traverse the *a*_*1*_/*a*_*2*_ band from the mid-point to the valley) we observe an increase in the prevalence (magnitude of the concavity) of this intermediate state when switching under positive bias; however, such a change is not evident when switching under negative bias.

Finally, the third feature map (Fig. 4i) once again shows increased activation within the *a*_*1*_/*a*_*2*_ bands; however, the gradient in activation goes in the opposite direction from the previous map (Fig. 4e), in that it is maximized near the valley boundary and decreases as we approach the middle of the band (Fig. 4j). Upon generating the resonance (Fig. 4k) and piezoresponse (Fig. 4l) loops, we observe an asymmetric resonance switching behavior, wherein near the valley boundary (i.e., high activation) hardening only occurs when switching under positive bias (marked 1). As we move towards the peak boundary (i.e., low activation), however, hardening during switching occurs under both positive and negative bias (green curves, Fig. 4h). An analogous trend in the piezoresponse concavity is observed (Fig. 4l), where the intermediate concavity is only observed when switching under positive bias when near the valley boundary (marked 1); yet, is observed under both positive and negative bias when near the peak boundary (green curve, marked 2).

### Phase-field simulations of ferroelectric switching

To understand the physical significance of the features identified by the autoencoder requires consideration of the domain structure, geometry, and switching processes. To guide our interpretation, we conducted phase-field switching studies (of a model film with a *c*/*a* domain structure) under a simulated tip bias (Methods, Supplementary Note 10, Supplementary Fig. 10). From these phase-field studies, we can observe the polarization as well as the local energetics during the switching process. Prior to the switching studies, the film exists in the up-poled state where the film has nearly uniform electrostatic energy.

**Fig. 5:**
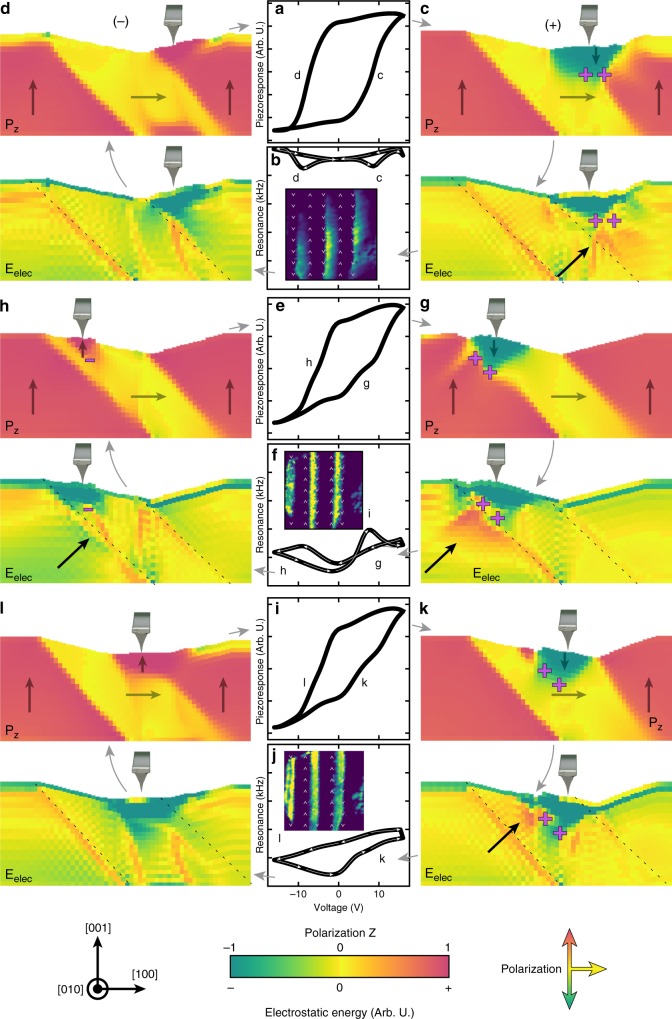
Interpretation of learned features based on phase-field simulations. **a**, **e**, **i** Generated piezoelectric hysteresis loops and **b**, **f**, **j** resonance response hysteresis loops under high activation as indicated in the activation maps the inset of **b**, **f**, **j**. These insets have the same colorscales as shown in Fig. 4. Arrows indicate the resonance pathway taken during switching. Phase-field simulations of (top) out-of-plane polarization, (bottom) electrostatic energy of switching under locally applied tip bias when the tip is positioned **c**, **d** within the *c* domain at the valley boundary, **g**, **h** within the *a* domain at the peak boundary, and **k**, **l** within the *a* domain at the valley boundary, wherein the applied tip bias is positive and negative at each position, respectively. Crystallographic orientation is indicated in the bottom left corner. Polarization direction is indicated in the right corner. Color scale for the polarization and electrostatic energy is presented as diverging perceptually uniform colormaps as indicated in the colorbar

Starting with the first “learned” feature, which is most pronounced when the tip is within the *c* domains near the valley boundary, we observe a square piezoelectric hysteresis loop (Fig. 5a) and an increase in the resonance frequency of the cantilever (i.e., an increase in the elastic modulus, Fig. 5b). From the initial state, phase-field simulations reveal that switching at this position results in the nucleation of a down-poled domain (Fig. 5c, top, Supplementary Movie 6). From the electrostatic energy, we observe a region of increased energy below the newly nucleated domain (Fig. 5c, arrow) as a result of the head-to-head charged domain wall which exists between the nucleated domain and the *a* domain. This electrostatic repulsion, from the growing charged domain wall, increases the local modulus of the material, which manifests as an increase in the cantilever resonance frequency. When applying negative bias to the tip, the bias reinforces the as-poled domain structure resulting in an unremarkable change in both the domain structure and local electrostatic energy (Fig. 5d).

Shifting our attention to the second “learned” feature, which is most pronounced at the peak of the *a* domains, where we observe intermediate concavities in the piezoresponse hysteresis loop (Fig. 5e) and elastic hardening when switching under positive and negative bias (Fig. 5f). Phase-field simulations reveal that switching at this location, under positive bias, results in the nucleation of a down-poled domain (Fig. 5g, top, Supplementary Movie 7) with a head-to-head positively charged domain wall. Turning our attention to the electrostatic energy we observe, as expected, a significant increase in the electrostatic energy near this charged domain wall (Fig. 5g, bottom, and indicated by the arrow). Thus, as the tip bias is increased, the newly nucleated domain grows laterally along the in-plane [100] and [010] resulting in an increase in the charged-domain-wall area. This, in turn, increases the stiffness, which manifests as an elastic hardening step. This hardening process continues until the finite volume probed by the tip is purely *c*-like, upon which application of further bias results in softening to saturation. When applying negative bias to the tip at this location, we observe the nucleation of an up-poled domain within the *a* domain (Fig. 5h, top). This up-poled domain must form a tail-to-tail negatively charged domain wall. In turn, the presence of the charged domain wall results in an increase in electrostatic energy (arrow, Fig. 5h, bottom) near this charged domain wall, which results in elastic hardening and softening to saturation with an identical mechanism as described for the behavior when switching under positive bias.

Finally, focusing on the third “learned” feature, which is most pronounced within the *a* domain near the valley, we observed intermediate concavities in the piezoresponse hysteresis loop (Fig. 5i) and elastic hardening when switching only under positive bias (Fig. 5j). Looking at the phase-field simulations under positive tip bias (Fig. 5k, Supplementary Movie 8), we observe the nucleation of a down-poled domain which is identical in form to that observed near the peak boundary within the *a* domain. As expected, the positive-bias branch of the piezoresponse loop and the resonance response have a similar form to the switching observed near the *a*-domain boundary, wherein the formation of a charged domain wall (Fig. 5k, bottom, arrow) results in an intermediate step in the piezoresponse loop and elastic hardening. When switching under negative bias, phase-field simulations reveal a different switching mechanism, wherein application of bias results in the expansion of the up-poled domain into the *a* domain, however, due to the geometry the domain is nominally uncharged (Fig. 5l). As a result, there is no evidence of either an intermediate concavity in the piezoresponse hysteresis loop nor a hardening step. All told, by interpreting the features learned by the autoencoder in the context of the domain structure and switching process we are able to identify features in both the piezoresponse hysteresis loops and resonance response related to the formation of charged domain walls during the switching process, which were not identified using conventional and linear machine learning analysis approaches.

## Discussion

In summary, we demonstrate how a deep sequence-to-sequence, LSTM autoencoder can be used to “learn” characteristic mechanisms of response from multichannel hyperspectral BEPS which are overlooked by classical machine-learning approaches. We train a sparse LSTM autoencoder capable of identifying characteristic materials responses and quantify their relative significance with spatial resolution. The increased aptitude of this methodology stems from the inclusion of sequential dependence inherent in the data. Specifically, using this approach we experimentally measure and visualize changes in the elastic modulus associated with nucleation and growth of charged domain walls which form as a result of the hierarchal domain geometry in tensile-strained PbZr_0.2_Ti_0.8_O_3_ thin films. This new capability provides a process to quantify subtle differences in switching mechanisms, providing a route towards the fully-automated and feedback-controlled nanoscale manipulation of domain structures and domain-wall geometries. Furthermore, this work establishes a new methodology capable of learning mechanisms of response from multichannel hyperspectral imagery which could be used for a range of existing and emerging hyperspectral imaging modalities in nanoscience.

More broadly, this work demonstrates a pathway towards the use of statistical approaches which take seemingly inconsequential data and, through mathematical transformations, creates observations from which meaning can be deduced. While such approaches have been used, most of these approaches have neglected the physical structure (e.g., symmetry, time, etc.) which provides a foundational basis for the data and the information which it contains. We emphasize how the sequential dependence of experimental data can be incorporated in such models, analogous concepts could be applied to include spatial position, symmetry, and both long- and short-range order, increasing their aptitude in inducing meaningful inference regarding materials systems and processes. This work paves the way for spectroscopic techniques wherein the conventional scientific methods of designing targeted experiments aimed at a specific hypothesis are supplanted by approaches which collect all seemingly relevant data, which can then be used to identify a hypothesis for empirical testing. Furthermore, this ability to automate the formation of fingerprints of physical processes from multichannel spectroscopies provides increased capabilities towards the controlled manipulation of structure–properties in an automated and self-regulating manner not possible with human-in-the-loop processes.

## Methods

### Growth of epitaxial PbZr_0.2_Ti_0.8_O_3_ thin films

Four hundred-nm-thick PbZr_0.2_Ti_0.8_O_3_ thin films were synthesized using pulsed-laser deposition by ablating a ceramic target of Pb_1.1_Zr_0.2_Ti_0.8_O_3_ using a KrF excimer laser (248 nm, LPX 305; Coherent), in an on-axis geometry with a 60 mm target-to-substrate spacing. The PbZr_0.2_Ti_0.8_O_3_ films were grown on 30 nm Ba_0.5_Sr_0.5_RuO_3_-buffered NdScO_3_ (110) single-crystal substrates which were affixed to the heater using Ag paint. The Ba_0.5_Sr_0.5_RuO_3_ bottom electrodes were grown at a heater temperature of 750 °C in a dynamic oxygen pressure of 20 mTorr, by ablating a ceramic Ba_0.5_Sr_0.5_RuO_3_ target (Praxair) at a laser fluence and a repetition rate of 1.8 J/cm^2^ and 2 Hz, respectively. The PbZr_0.2_Ti_0.8_O_3_ films were grown at a heater temperature of 600 °C in a dynamic oxygen pressure of 50 mTorr, with a laser fluence and repetition frequency of 1.9 J/cm2 and 14 Hz, respectively. Following growth, all heterostructures were cooled to room temperature in a static oxygen pressure of 760 Torr at 5 °C/min.

### Band-excitation piezoresponse spectroscopy

BEPS studies were performed at the Center for Nanophase Materials Science (CNMS) at Oak Ridge National Laboratory (ORNL) using a custom Cypher (Asylum Research) atomic force microscope controlled with a Labview- and Matlab-based controller. A bipolar-triangular-switching waveform was applied using a conductive scanning-probe tip in a square grid measuring the cantilever response caused by the band-excitation waveform in the time domain. Following processing with a fast-Fourier transform, the cantilever resonance response was fit to a simple harmonic oscillator model, allowing the extraction of piezoresponse amplitude, phase, cantilever resonance frequency, and dissipation. The use of band excitation for these measurements is crucial as it minimizes effects from changing tip–sample contact resonances that can alter the observed response, enabling consistent measurements of piezoresponse throughout multiple dimensions (that is, frequency, spatial, voltage, time, and so on; Supplementary Note 3). All measurements were carried out using Pt/Ir-coated probe tips (NanoSensor PPP-EFM). Switching spectroscopy measurements were measured at a resonance frequency of ~132 kHz (with a bandwidth of 60 kHz). The DC voltage was chosen such that the piezoelectric hysteresis loops were saturated in both the positive and negative direction. The local piezoresponse was measured at remanence (following a dwell time of 0.5 ms), with a BE waveform of sinc character (peak-to-peak voltage of 1 V).

### Neural-network structure and training

The LSTM RNN autoencoders were built in Keras using the Tensorflow backend. The network trained on the piezoresponse data had four encoding and decoding layers each of size 128. Dropout within the encoding and decoding layer was fixed at 20%. The low-dimensional embedding layer had a size of 16 and *l*_1_ regularization (*λ* = 1 × 10^−5^). Batch-normalization layers were included prior to and following the low-dimensional embedding layer. The network was trained using Adam as an optimizer with an initial learning rate (*L* = 3 × 10^−5^) for 16,000 epochs. For the analysis of the resonance data the network used was identical to the network used for the piezoresponse data with the exception that the network was trained for 22,000 epochs. Training was completed using a local workstation equipped with a NVIDIA Titan X graphics processing unit (GPU) or on the Savio supercomputer cluster equipped with GPU nodes with NVIDIA K80 GPUs. To accelerate the training of the generated responses formed, after training the autoencoder sufficiently, the weights through the low-dimensional layer were fixed and the decoder was trained for one million epochs without dropout.

### Phase-field simulations

A three-dimensional model was applied to simulate the evolution of ferroelectric polarizations $\left( {P_i\;(i = 1,2,3)} \right)$ of the PbZr_0.2_Ti_0.8_O_3_ (PZT) thin film by numerically solving the time-dependent Landau–Ginzburg–Devonshire (LGD) equations:^64^

1 $$\frac{{\partial P_i\left( {x,t} \right)}}{{\partial t}} = - L\frac{{\delta F}}{{\delta P_i\left( {x,t} \right)}},\;i = 1,\;2,\;3$$

in which _*Pi*_ is the polarization vector, *x* is the spatial position, *t* is the time, *L* is the kinetic coefficient related to the domain wall mobility, and *F* is the total free energy as shown below:^65^

2 $$F = {\int}_V {\left[ {f_{{\mathrm{Land}}}\left( {P_i} \right) + f_{{\mathrm{Grad}}}\left( {P_{i,j}} \right) + f_{{\mathrm{Elas}}}\left( {P_i,\varepsilon _{ij}} \right) + f_{{\mathrm{Elec}}}\left( {P_i,E_i} \right)} \right]{\mathrm{d}}V}$$

in which $f_{{\mathrm{Land}}}\left( {P_i} \right)$, $f_{{\mathrm{Grad}}}\left( {P_{i,j}} \right),f_{{\mathrm{Elas}}}\left( {P_i,\varepsilon _{ij}} \right),f_{{\mathrm{Elec}}}\left( {P_i,E_i} \right)$ represent the LGD free energy density, gradient energy density, elastic energy density, and electrostatic energy density, respectively. Details of these energy density terms as well as the coefficients related to these energy terms are collected from literature.^66^ Here we adopt a sixth-order polynomial expansion of _*Pi*_ for $f_{{\mathrm{Land}}}\left( {P_i} \right)$, and choose the dielectric constant to be *κ* = 50 for PZT. The gradient energy coefficients are set to be *G*_11_/*G*_110_ = 0.6, where *G*_110_ = 1.73 × 10^−10^ C^−2^ m^4^ N.^67^ The simulation size is a realistic three-dimensional geometry sampled on a fine grid mesh of 128Δ*x* × 128Δ*x* × 32Δ*x*, where the grid size Δ*x* *=* 1.0 nm. The film and substrate thickness are 20Δ*x* and 10Δ*x*, respectively. A semi-implicit spectral method^68^ is used to solve the time-dependent LGD equation, with periodic boundary conditions applied in *x*_1_ and *x*_2_ directions, and thin film boundary conditions applied in *x*_3_ direction. The initial structure consists of (100)_*a*_/(001)_*c*_ preset domain structure. The entire thin film is subjected to a homogeneous 0.3% tensile strain by the substrate. Electric bias is modeled using a Lorentz function $\varphi \left( {x,y} \right) = \frac{{\varphi _0\gamma ^2}}{{\left( {r - a} \right)^2 + \gamma ^2}}$ where *r* is the distance from the tip and *γ* is the half-width at half-maximum (HWHM) of applied bias $\left( {\varphi _0} \right)$. The tips are located near the *c*/*a* domain boundaries as described in the main text. The average polarization in a 5Δ*x* × 5Δ*x* × 6Δ*x* cuboid volume near the tip center is collected to calculate the hysteresis loop.

## Supplementary information

Supplementary Information

Description of Additional Supplementary Files

Supplementary_Movie_1_BE_Switching

Supplementary_Movie_2_Piezoresponse_training_movie

Supplementary_Movie_3_resonance_training_movie

Supplementary_Movie_4_Piezoresponse_Generator_movie

Supplementary_Movie_5_Resonance_Generator_movie

Supplementary_Movie_6_Switching_movie_tip4

Supplementary_Movie_7_Switching_movie_tip2

Supplementary_Movie_8_Switching_movie_tip3

## Data Availability

All data and analysis code is made available under the BSD 3-clause license at https://github.com/jagar2/Revealing-Ferroelectric-Switching-Character-Using-Deep-Recurrent-Neural-Networks. The source code can be obtained and cited using 10.5281/zenodo.3405660. The raw data can be obtained and cited using 10.5281/zenodo.1482091. This manuscript is also available as an executable Jupyter paper at https://bit.ly/2nCLGDC.
