# Supplementary material for: Revealing ferroelectric switching character using deep recurrent neural networks
Source: Nat Commun. 2019 Oct 22;10:4809. doi: 10.1038/s41467-019-12750-0 (PMC6805893; doi:10.1038/s41467-019-12750-0)
Supplement: Supplementary file 1 — Supplementary Information [file 41467_2019_12750_MOESM1_ESM.pdf]

Supplementary Information

Revealing Ferroelectric Switching Character Using Deep Recurrent Neural Networks

Joshua C. Agar et al.

Supplementary Note 1: Accessing the Jupyter Notebook

All codes, pre-trained networks, and analysis are available as an executable Jupyter notebook. For those who are familiar with Jupyter and git the repository and the data can be downloaded by typing in the command line:

```
git clone https://github.com/jagar2/Revealing-Ferroelectric-Switching-Character-Using-Deep-Recurrent-Neural-Networks.git
```

Alternatively, the notebook can be run on the cloud using Google Collaboratory. Instruction on how to run the codes using this method are provided at: https://github.com/jagar2/Revealing-Ferroelectric-Switching-Character-Using-Deep-Recurrent-Neural-Networks. Note for this option the only requirement is a Google account with access to a Google Drive. No software is needed. Using Google Collaboratory, you are provided free access to both graphics processing units (GPUs) and tensor processing units (TPUs) using these resources can greatly accelerate training of the neural networks if you do not have access to GPU accelerated computing.

Supplementary Note 2: Additional Structural Information

To support the structural studies provided in the manuscript we provide a modified temperature-strain phase diagram for $PbZr_{0.2}Ti_{0.8}O_3$. Based on this diagram, at room temperature the film should exist with a monodomain *c* domain structure under large compressive strain. As the compressive strain decreases, it is energetically favorable to accommodate some in-plane oriented *a* domains. As the strain becomes more tensile in nature the domain structure transitions to a purely in-plane oriented a_1/a_2 domain structure. In this work, we have grown films at ~1.7% tensile strain, at this intermediate strain there is competition between *c*/*a* and a_1/a_2 domain structures, which in turn gives rise to the hierarchical domain structure observed.

Additionally, detailed reciprocal space mapping studies were conducted about the 220- and 002-diffraction condition of the substrate and film, respectively (b). From these studies, we observe two sets of five peaks each. The first set of five peaks has out-of-plane lattice parameters nearly identical to what is expected for bulk $PbZr_{0.2}Ti_{0.8}O_3$ (~4.129 Å). There is a central peak

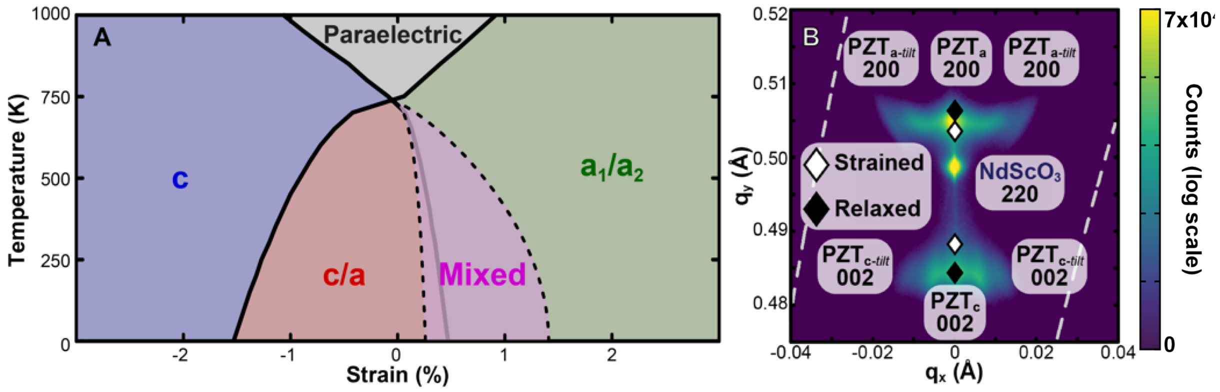

Supplementary Figure 1 | Additional structural studies. a, Temperature-strain phase diagram for $PbZr_{0.2}Ti_{0.8}O_3$. **b,** Symmetric reciprocal space map of the 400 nm thick $PbZr_{0.2}Ti_{0.8}O_3$/20 nm $Ba_{0.5}Sr_{0.5}$-RuO_3/$NdScO_3$ (110) heterostructures. Maps obtained around the substrate 220 diffraction condition. Coherently strained and relaxed peak positions are indicated.

aligned with the out-of-plane axis of the substrate (PZT_c} 002), and two sets of tilted reflections ($PZT_{c\text{-}tilt}$ 002). The first set of tilted reflections are tilted 0.6° off axis, whereas, the second set of peaks are tilted by 1.2° relative to the [001]. The second set of five peaks has an out-of-plane lattice parameter of ~3.96 Å, nearly halfway in between the coherently strained a relaxed *a* domain peak position. Once again there is a central peak aligned with the substrate normal (PZT_a 200), surrounded by two sets of peaks ($PZT_{a\text{-}tilt}$ 200) with varying tilts. The first set of reflections have a moderate tilt of ~1°, whereas, the second set of peaks has a more significant tilt of ~1.8° relative to the [001].

Supplementary Note 3: Band Excitation Piezoresponse Force Microscopy

Traditional approaches to scanning probe microscopy and, in particular PFM, tend to rely on periodically exciting a cantilever using a single-frequency excitation at, or very near to, the cantilever resonance to perturb the sample, invoking a response in the cantilever amplified by its resonance. In single-frequency excitation, the response of the cantilever, and ultimately the signal of interest, is measured using a lock-in amplifier[1]. While powerful for imaging highly-responsive materials with large domain features, the limitations of a single-frequency approach become obvious even when considering a simple idealized model of the cantilever resonance in the form of a simple harmonic oscillator (SHO). In this case, the response of the cantilever is primarily determined by the resonance frequency (defined by the tip-surface spring constant), amplitude (a measure of the response), and quality factor of cantilever resonance (i.e., tip-surface dissipation), all of which are convoluted by the tip-surface interaction[2]. While more advanced approaches based

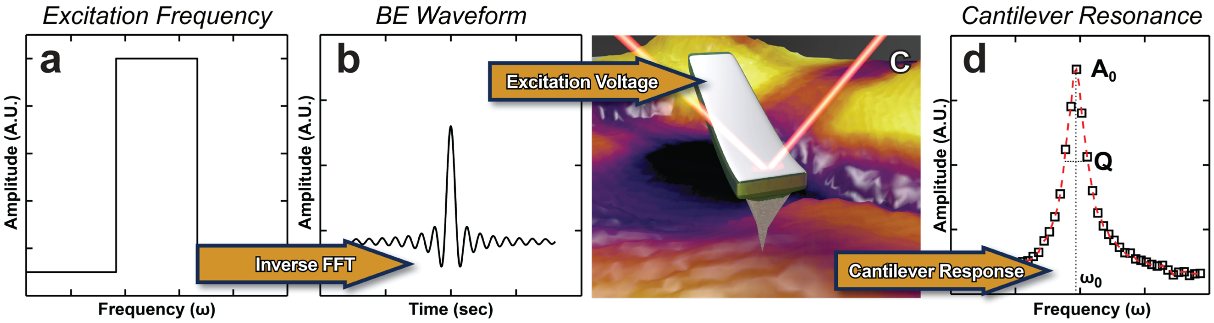

Supplementary Figure 1 | Schematic illustration of workflow for band-excitation piezoresponse force microscopy (BE-PFM) imaging. a, Typical frequency domain plot of BE signal chosen to excite the cantilever near the cantilever resonance. **b,** Example BE-excitation sinc waveform in time domain used to excite the tip at a range of frequencies. **c,** Schematic drawing of AFM cantilever in contact with surface. **d,** Typical cantilever resonance response shown in frequency domain. Red-dashed line shows the fit, based on equations 1-2. Characteristic variables which define the cantilever/material response are indicated.

on phase-locked loops (which use external circuitry to try to maintain the system at resonance) or dual-frequency resonance tracking (which excites, measures, and then tracks the resonance using two frequencies) provides a more accurate measure of piezoresponse, they still only provide minor improvements applicable under the strictest set of assumptions[3].

To overcome these bandwidth limitations and accurately measure piezoresponse without artifacts imposed by changing cantilever dynamics it is crucial to measure the cantilever response over a large bandwidth near the cantilever resonance (Supplementary Figure 2a). To do this, we used band-excitation piezoresponse force microscopy (BE-PFM), which uses a computer-generated waveform (Supplementary Figure 2b) spanning band of frequencies near the measured cantilever resonance to electrically perturb the material (Supplementary Figure 2c)[2]. The response of the cantilever can then be measured with a high-speed data acquisition system and subsequently Fourier transformed (Supplementary Figure 2d) into the frequency domain. Following data collection, assuming that the tip-sample interaction is weak, the amplitude and phase can be fit to a SHO model as described in equations 1-2, where A_0 and ω_0 are the amplitude and frequency at resonance.

$$\boldsymbol{A(\omega)=\frac{A_0\omega_0^2}{\sqrt{\left(\omega^2-\omega_0^2\right)^2+(\omega\omega_0/Q)^2}}} \quad (1)$$

$$\boldsymbol{\tan\left(\theta(\omega)\right)=\frac{\omega\omega_0/Q}{\omega^2-\omega_0^2}} \quad (2)$$

Following fitting (Supplementary Figure 2d), the error of the fits can be evaluated (*i.e.*, the quality of the deconvolution), and if good, the data yields (x,y) maps of resonance amplitude (A_0), resonance frequency (ω_0), and quality factor (Q) as well as the phase (θ) of the response. Therefore, with the proper care, the application of BE-PFM enables the exclusion of cross-talk (associated with position dependent changes in the cantilever resonance) minimizing the contribution from the tip-surface interaction.

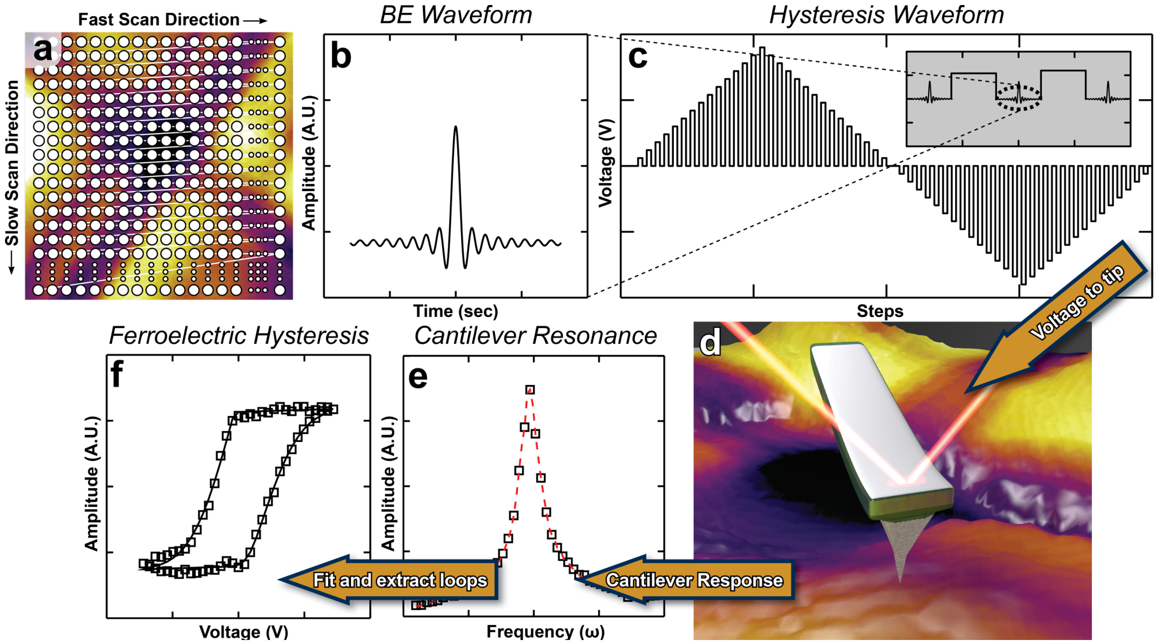

Supplementary Figure 2 | Schematic illustration of workflow for band excitation piezoresponse spectroscopy measurements (BEPS). a, Schematic of the sampling, and fast and slow scan direction used for imaging. **b,** Example BE chirp waveform in time domain used to excite the tip at a range of frequencies. **c,** Triangular switching waveform used to locally switch the film. **d,** Schematic drawing of AFM cantilever in contact with surface. **e,** Typical cantilever resonance response shown in frequency domain. Red dashed line shows the fit, based on equations 1-2. **f,** Typical piezoelectric hysteresis loop obtained from BEPS.

Building off the BE-based imaging technique, it is possible to add additional dimensionality to such measurements, providing deeper insight into the response of the material. Specifically, instead of just measuring $\{A,\theta\}(x,y,\omega)$ we can add an additional dc-voltage dimensionality to the measurement [that is, $\{A,\theta\}(x,y,\omega,V_{dc})$] enabling the measurement of local piezoelectric hysteresis loops while taking advantage of the enhanced measurement precision provided by band excitation. To do this, we superimposed a n x n grid, on a previously scanned region of interest (Supplementary Figure 3a). At each point, a full bipolar triangular switching waveform is applied to the cantilever (Supplementary Figure 3b-d) and readout is conducted in the off-state (that is, remanent state) by superimposing a band-excitation waveform (sense pulse). This process happens rapidly (total elapsed time <5 ms). Following data acquisition, the data is fit using a SHO model as previously described (Supplementary Figure 3e), yielding data of the form $\{A_0, \omega_0, Q, \theta\}(x, y, V_{dc})$. By optimizing the rotation angle (φ) to maximize the real component of the hysteresis loop mixed-signal ($A_0 \cos\varphi$) it is possible to generate local piezoelectric hysteresis loops of the same general form as typical macroscopic ferroelectric hysteresis loops (Supplementary Figure 3f).

It is worth emphasizing that all units are intentionally reported in arbitrary units. To measure the deflection of the cantilever it is standard practice to use the beam bounce approach. In this approach, a laser is reflected off the back of the cantilever and the *angular displacement*, not the *vertical displacement*, is measured by a quadrant photodiode. To accurately convert the angular dependence into a displacement requires a number of assumptions to be made about the cantilever mode shape. While these assumptions are fairly accurate in air, they deviate from ideality when the cantilever is in contact with the surface. In turn, the measured effective piezoresponse using

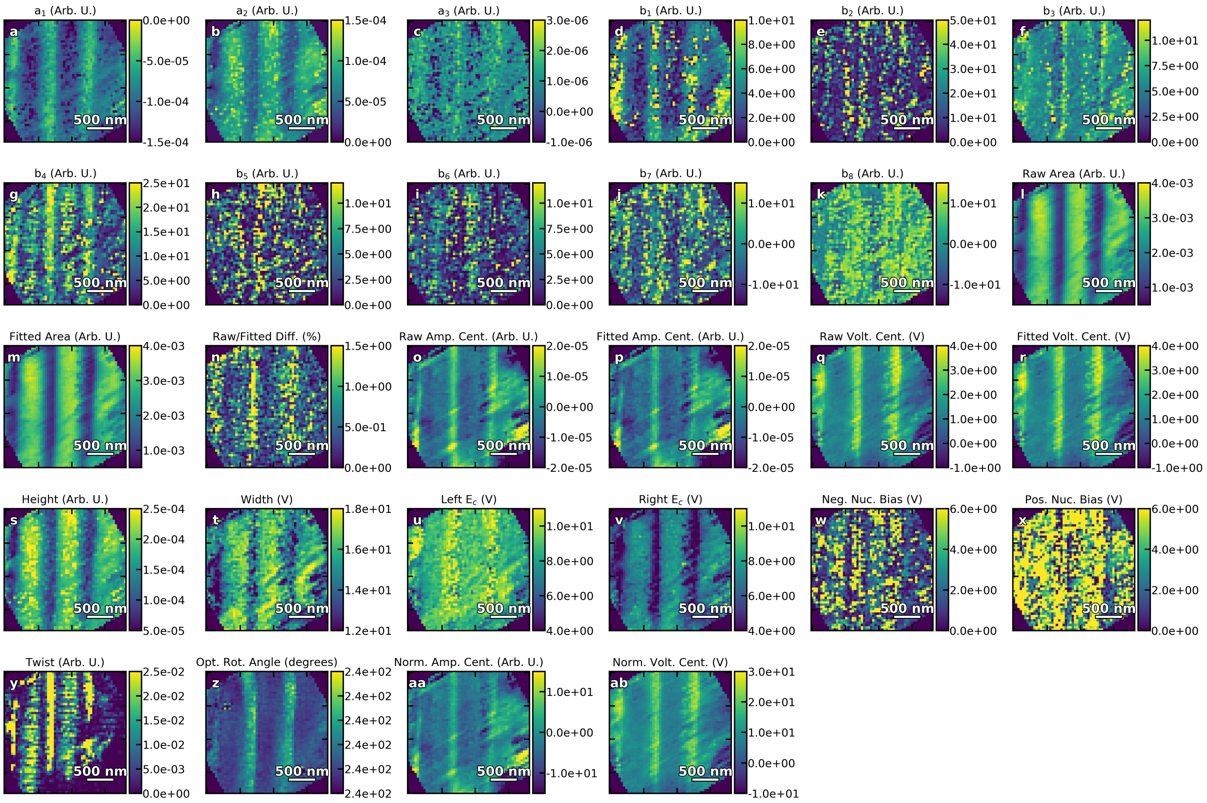

Supplementary Figure 4 | Spatial maps of loop fitting parameters obtained from band excitation switching spectroscopy of $PbZr_{0.2}Ti_{0.8}O_3$ with hierarchical domain structures.

the beam bounce approach can easily vary by orders of magnitude depending on the laser spot position and contact resonance frequency[4]. Such values of piezoresponse commonly reported in literature generally do not include consideration for these effects (unless specifically mentioned) and thus should be considered as being reported in arbitrary units.

Supplementary Note 4: Loop Fitting Piezoresponse Loops

Spatial maps of the various loop fitting parameters obtained from loop fitting are provided (supplementary Fig. 4). While the major characteristic features of the loops are extracted using this approach these maps provide limited insights into the subtitle differences in switching behavior which underpin the physical response. Additionally, in the exploration of the quality of the loop-fitting results, it is apparent that the function lacks the complexity to fit the varied

response types observed. The addition of more parameters to the fitting function could resolve this issue, however, would likely results in overfitting.

Supplementary Note 5: Decomposition Algorithms – Principal Component Analysis

One common approach used to visualize statistical variance in high-dimensional data (*e.g.*, BEPS) is principal component analysis (PCA). PCA is a statistical method that converts a set of observations (in this case piezoresponse values at each voltage), to a set of principal components which are linearly uncorrelated (orthogonal to each other). In simple terms, this approach finds perpendicular directions in the data volume of maximal variance, such that the data can be projected onto lines in those direction with minimal loss of information. These principal components are ranked in the order of the variance of the data which they represent. Thus, if the data is highly-correlated, nearly all the information of the dataset can be represented by a subset of the highest-ranked principal components. These principal components can be used to identify

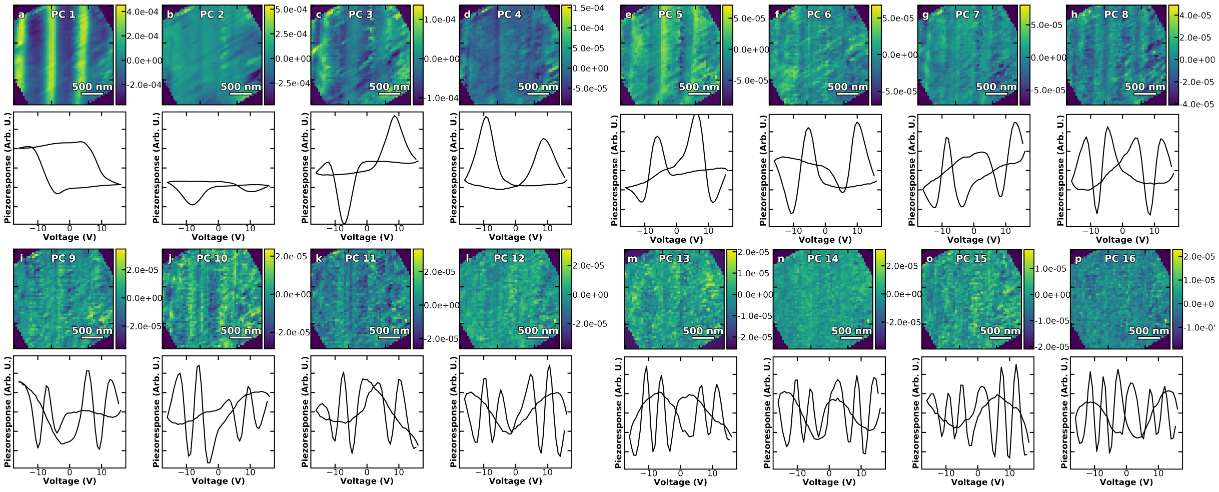

Supplementary Figure 5 | Principal component analysis of piezoresponse hysteresis loops obtained from band excitation piezoresponse force microscopy of 400 nm $PbZr_{0.2}Ti_{0.8}O_3$ films with hierarchical domain structures. Figures on the top show the loading maps (eigenvectors) and bottom shows the principal components (eigenvalues).

correlations in data set by themselves, however, have no physical significance and thus are difficult to interpret. Since the principal components are ranked in the order of the variance in the data, most of the information in the data set is contained within the higher-ranking principal components; whereas, the lower-ranking principal components contain uncorrelated noise.

To represent this, we show the eigenvalues (top) and eigenvectors (bottom) of the first 16 principal components (supplementary Fig. 5) of the piezoelectric hysteresis loops. Looking at the first eigenvalue map, it is evident that the majority of the information is represented by this first principal component and that this component has a response similar to the piezoelectric hysteresis loops. As we look at the higher-order principal components, we notice a reduction in the visual similarity between the loading maps and the film structure. The principal components beyond the first principal component have recognizable features but are increasingly complex and thus are difficult to interpret. Beyond the first few principal components, the resulting principal components have increased complexity and the loading maps show reduced correlations to the domain structure. This indicates they are representing uncorrelated information or noise, and therefore are of minimal significance. Additional analysis of other signal channels is provided (Jupyter Notebook J9-13)

Supplementary Note 6: Decomposition Algorithms – Non-negative Matrix Factorization

A complementary decomposition algorithm which has seen recent use in the analysis of BEPS data is non-negative matrix factorization (NMF). NMF has gained favor because of its ability to extract non-negative (*i.e.* all positive abundance maps) sparse representations of data which are easier to interpret than those obtained from PCA. NMF works by finding a decomposition of positive samples X into two matrices W the spectral signature matrix and H the abundance matrix where all of the elements are non-negative. This is accomplished by optimizing the distance d

between X and the matrix product $W\,H$ using the Frobenius norm (eq. 3-4). To improve the generalization of the optimization regularization is added to the loss function, typical the loss function allows for control of the regularization strength with (α) and the l_1 and l_2 character of the regularization using the l_1-ratio (ρ, eq. 4).

$$d_{Fro}(\mathbf{X},\mathbf{Y}) = \frac{1}{2}\|X - WH\|_{Fro}^2 = \frac{1}{2}\sum_{i,j}\left(X_{ij} - W_{ij}H_{ij}\right)^2 \tag{3}$$

$$\alpha\rho\|W\|_1 + \alpha\rho\|H\|_1 + \frac{\alpha(1-\rho)}{2}\|W\|_{Fro}^2 + \frac{\alpha(1-\rho)}{2}\|H\|_{Fro}^2 \tag{4}$$

To demonstrate this approach, we conducted NMF on the raw piezoresponse hysteresis loops wherein the hysteresis loops were shifted to have all non-negative values. In this example we computed the NMF assuming the number of components $n = 4$, with $\alpha = 1\text{x}10^{-7}$, and an l_1-ratio = 1 to impose sparsity. We show abundance maps (top) and spectral endmembers (bottom) obtained from NMF. From these maps we again as expected see features resembling the underlying domain structure. Specifically we observe abundance maps which highlight the c (a) domains

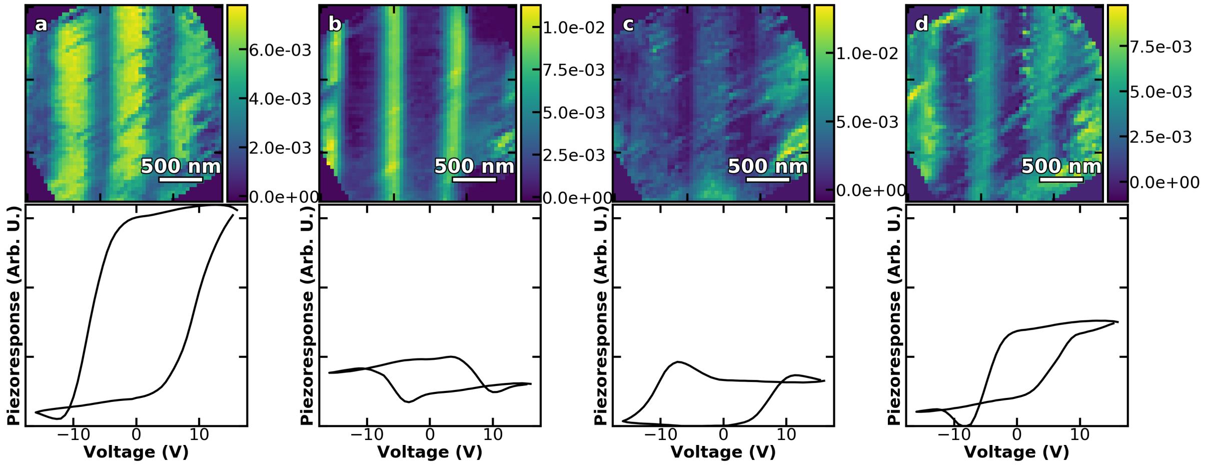

Supplementary Figure 6 | Non-Negative matrix factorization of piezoresponse hysteresis loops obtained from band excitation piezoresponse force microscopy of 400 nm $PbZr_{0.2}Ti_{0.8}O_3$ films with hierarchical domain structures. Figures on the top show the abundance maps and bottom shows the endmember.

(supplementary Fig. 6a-b, top respectively) and maps which highlight some local variances in the response predominately in the *c/a* (a_1/a_2) domains (supplementary Fig. 6c-d, top respectively). Changing our attention to the endmember spectra we first notice a response which looks like a classical piezoelectric hysteresis loop (supplementary Fig. 6a, bottom), however, the other endmember components while having some resemblance to a hysteresis loops have a non-classical shape which is difficult to interpret (supplementary Fig. 6d, bottom). This complication in the interpretability of the results is due to the inability of the algorithm to impose perfect sparsity and thus the response is a linear combination of multiple endmembers. Additional analysis of other signal channels is provided (Jupyter Notebook J14-18).

Supplementary Note 7: Clustering Algorithms

Another common approach to distill information from BEPS data is to apply clustering algorithms to group the spectra based on the statistical variance in the response. In this work, we applied the scikit-learn implementation of *k*-means clustering[5]. Specifically, *k*-means clustering partitions *n* samples into *k* groups of equal variances by minimizing the within-cluster sum-of-

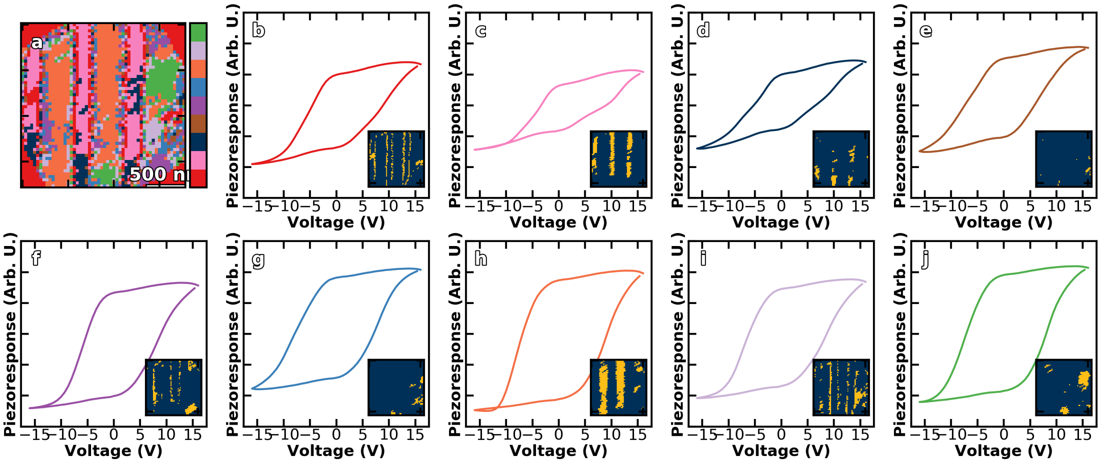

Supplementary Figure 7 | Divisive clustering of piezoresponse hysteresis loops obtained from band excitation piezoresponse force microscopy of 400 nm $PbZr_{0.2}Ti_{0.8}O_3$ films with hierarchical domain structures. a, Clustering map, **b,** average piezoelectric hysteresis loop within specific cluster (identified

squares. The algorithm achieves this by randomly initializing *k* clusters, followed by looping through two steps. The first assigns each sample to its nearest centroid. In the second step the centroid position is updated based on the mean of the previously assigned centroid. This process continues until the difference between the old and new centroids reaches some threshold value. Generally, it is not known *a priori* what the number of clusters *k* is, however, in the case of the hierarchical domain structure $PbZr_{0.2}Ti_{0.8}O_3$ we know there are discrete *c*/*a* and a_1/a_2 bands. In turn, we applied divisive *k* means clustering wherein we first clustered the data with $k = 2$ isolating the *c*/*a* and a_1/a_2 domain bands. The identified regions were then clustered where *k* was varied to form spatially continuous clusters which could be interpreted based on our understanding of the domain structure. Most simply, *k*-means clustering can be applied directly to the raw piezoelectric hysteresis loops wherein each voltage step is used as an independent sample in high dimensional space. While this approach is able to identify the *c*/*a* and a_1/a_2 bands further clustering results in no further insight (supplementary Fig. 7). For completeness, we explored other clustering methodologies (*see* Jupyter notebook Figure J19-37).

Supplementary Note 8: Long Short-Term Memory Neuron Structure

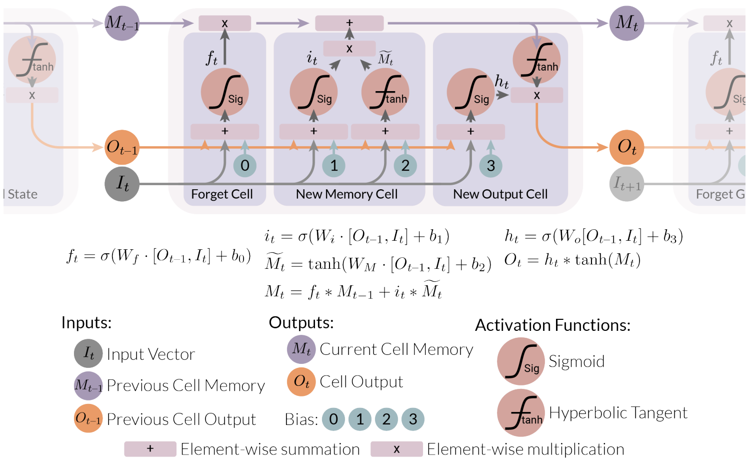

Supplementary Figure 8 | Schematic drawing showing the workflow of a long-short term memory neuron. The equations used for each neuron is indicated in the diagram. Different components of the neuron is indicated graphically.

In dealing with data that has a sequential or temporal dependence (*e.g.* BEPS) it is important that a neural network architecture can consider this temporal dependence. The most effective approaches have been to utilize some form of a gated recurrent neural network (RNN). These networks propagate through time while having a leaky unit which allows information to be passed between time steps without causing the gradient to explode or vanish, in doing so accumulating information about the data over a long duration in time. One particularly common implementation of a gated RNN is called a long short-term memory (LSTM) RNN (supplementary Fig. 8). LSTM in addition to the memory gate within its substructure has a second learned gate which controls when to reset or forget the memory state. In more detail, LSTM's have a triptych cell structure. The first cell, the forget cell takes as an input the output from the previous time step and the current input. Within the forget gate there is an internal neuron which multiplies the inputs by two weight matrices of size (N_i x N_{ln} and N_{ln} x N_{ln}) where N_i is the number of inputs from the previous layer and N_{ln} is the size of the current layer. The element-wise summation of the result plus a bias b_0 of size N_{ln} is passed through a sigmoid non-linearity resulting in a value between 0 and 1. This output from the forget gate (f_t, eq. 5) is element-wise multiplied by the previous memory cell state, in doing so, gating or controlling how much information from the previous memory state M_{t-1} is retained.

$$f_t = \sigma\left(W_f \cdot [O_{t-1}, I_t] + b_0\right) \tag{5}$$

The second cell within the LSTM neuron, the new memory cell builds information to pass to the next time step by having two internal neurons. The first, a neuron i_t (eq. 6), has the same form as the forget gate and acts to control the magnitude of the new cell state memory. The second neuron takes the same inputs and multiplies these values by two matrices of size (N_i x N_{ln} and N_{ln} x N_{ln}). Following the addition of a bias b_2 of size N_{ln}, the element-wise summation of the results is

then passed through a hyperbolic tangent non-linearity ($\widetilde{M_t}$, equation 7), which is multiplied by the new memory gate (i_t) and element-wise added to the previous cells memory (M_{t-1}) to form the new cell memory state (M_t, eq. 8).

$$i_t = \sigma(W_i \cdot [O_{t-1}, I_t] + b_1) \tag{6}$$

$$\widetilde{M_t} = \tanh(W_M \cdot [O_{t-1}, I_t] + b_2) \tag{7}$$

$$M_t = f_t * M_{t-1} + i_t * \widetilde{M_t} \tag{8}$$

The final cell, the *new output cell* forms the output for time step O_t by first computing an output gate of similar form to the previous gates (h_t, eq. 9). This gate controls what information from the cell state is passed as an output to the next layer in the model. Finally, the new cell state is passed through a hyperbolic tangent non-linearity (forcing the output to be between -1 and 1). This output is then multiplied in an element-wise fashion by the output cell gate (h_t) to form the output (O_t, eq. 10). In the networks forward pass is computed for each time step through time. In the training process, the gradient is calculated through time in a process known as backpropagation through time, optimizing each of the $4(N_{ln}^2 + N_I \times N_{ln})$ weights and N_{ln} biases per LSTM neuron.

$$h_t = \sigma(W_o[O_{t-1}, I_t] + b_3) \tag{9}$$

$$O_t = h_t * \mathrm{Tanh}(M_t) \tag{10}$$

Supplementary Note 9: Additional Details about the Autoencoder Architecture

In our description of the autoencoder architecture for brevity we excluded some of the subtitle details of the network structure. Here, we provide further details that can aid in the reproducibility of our method (supplementary Fig. 9). Initially, the network starts by taking as an input a mini-batch of response curves through time, where each voltage step is considered an independent time step. Generally, the mini-batches are chosen to be either ¼ of the data set or the maximum permitted by the graphics processing unit (GPU) on board memory. The input data is then passed

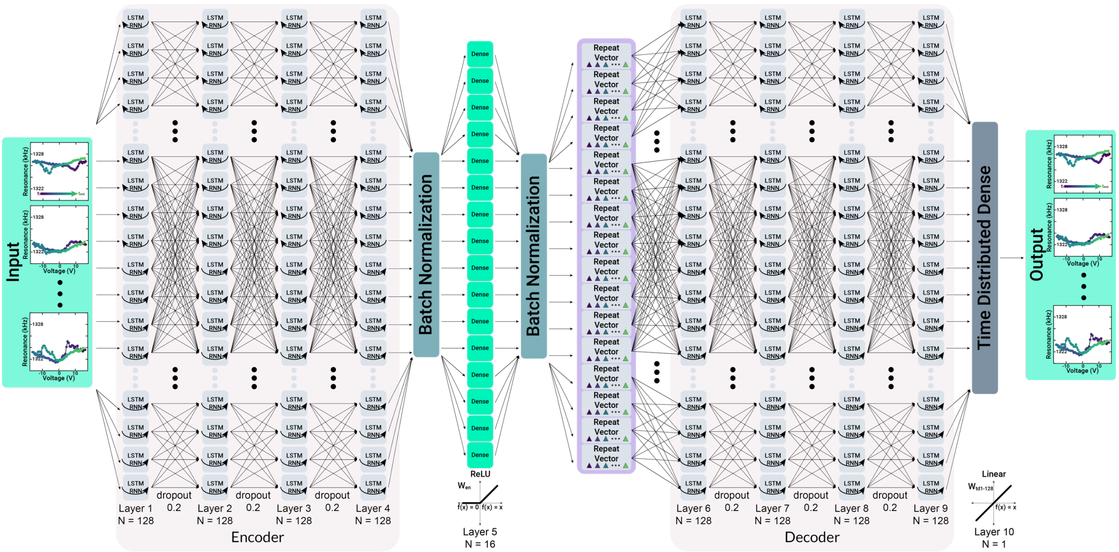

Supplementary Figure 9 | Schematic drawing showing a detailed view of the neural network architecture. Arrows indicate the passing of information between different layer. Specialty layers are indicated graphically.

to the first layer of the encoder. The encoding layer is composed of 128 LSTM neurons which function as described in *Section 8* of the supplementary information. Within these 128 LSTM neurons, half of the neurons propagate through the data in the forward direction (*i.e.*, from start-to-finish) and the other in the reverse direction (*i.e.*, from finish-to-start). The inclusion of bidirectionality of the network helps mitigate bias associated with the first timesteps in the sequence, which in turn, tends to give better results. In the encoder, there are four encoding layers, wherein between each layer dropout of 20% was applied. Dropout seeks to minimize co-adaptation in the network by randomly removing 20% of the network connections during training. Since the application of dropout acts to *thin* the network at test time the weights have to be adjusted based on average scaling to compensate for dropout. After the encoder, the output is batch normalized. Batch normalization tends to increase the stability of the neural network by adjusting the shift and the scale of the outputs by subtracting the *batch mean* and dividing by the *batch standard deviation*. This mathematical transformation cannot be done by simply subtracting the mean and

dividing by the standard deviation as this would merely increase the loss function in a way that would be erased by stochastic gradient descent. To solve this issue, two learnable parameters (γ, β) are used to scale the magnitude of the normalization such that the network can be optimized with inclusion of this normalization. More explicitly, batch normalization takes an input of values x from a mini-batch $\mathcal{B} = \{x_{1...m}\}$ to compute the batch normalized output $BN_{\gamma,\mathcal{B}}(x_i)$ as described by eq. 11-14, where x_i is a collection of samples, m is the number of samples, $\mu_{\mathcal{B}}$ is the batch mean, and $\sigma_{\mathcal{B}}^2$ is the batch variance.

$$\mu_{\mathcal{B}} = \frac{1}{m}\sum_{i=1}^{m} x_i \quad (11)$$

$$\sigma_{\mathcal{B}}^2 = \frac{1}{m}\sum_{i=1}^{m} (x_i - \mu_{\mathcal{B}})^2 \quad (12)$$

$$\widehat{x_i} = \frac{x_i - \mu_{\mathcal{B}}}{\sqrt{\sigma_{\mathcal{B}}^2 + \varepsilon}} \quad (13)$$

$$BN_{\gamma,\mathcal{B}}(x_i) = \gamma\widehat{x_i} + \beta \quad (14)$$

Following batch normalization, the output from the hidden layer is passed to the low-dimensional embedding layer. It is important to note that only the last time step in the data is passed to the low-dimensional layer. This reduces the dimensionality of the network such that each spectra is represented by only one value per neuron. Since the encoder is composed of LSTM neurons the final time step of the encoder has information regarding both the short- and long- term time dependencies in the data. This low-dimensional embedding layer is composed of sixteen neurons which are designed to impose sparsity. This is achieved using two synergistic approaches as described in the main text: First, we constrict the outputs to non-negative values by selecting a rectified-linear activation function ($f(x) = \max(0, x)$, ReLu). Secondly, we add strong l_1 regularization which adds an additional contribution to the loss function proportional to the sum

of the weights ($\lambda \sum_i |W_i|$) thus, only those activations which significantly improve the model's accuracy are non-zero. Following the low-dimensional embedding layer, a second batch normalization layer is applied. Immediately preceding the decoder, the output from the batch-normalization layer is repeated to the original vector length. This vector is then passed to the decoder which is constructed with an identical form as the encoder (*i.e.*, having 4 layers, each with 128 LSTM neurons). Following the decoder, the output is passed to a so-called time distributed layer. This layer has a single dense neuron with a linear activation function for each time step in the spectra. In turn, this layer converts the abstract information extracted from the decoder and converts it back to the original time series such that it can be optimized using stochastic gradient descent.

Supplementary Note 10: Phase-Field Simulations of Ferroelectric Switching

In addition to visualizing the switching process using phase-field simulations it is also possible to compute the local ferroelectric hysteresis loops by extracting the average polarization under the tip. Computation of the ferroelectric hysteresis loop at various tip locations (supplementary Fig. 10) reveals switching mechanism which have similarity to the observed piezoresponse hysteresis Specifically, we observe square loops when the tip is within the *c*/*a* band near the valley boundary (supplementary Fig. 10a), intermediate concavities in the piezoelectric hysteresis loop when switching under positive and negative bias when in the a_1/a_2 band near the valley boundary (supplementary Fig. 10b), and intermediate concavities only when switching under positive bias when within the a_1/a_2 boundary near the peak (supplementary Fig. 10c). This is the exact same

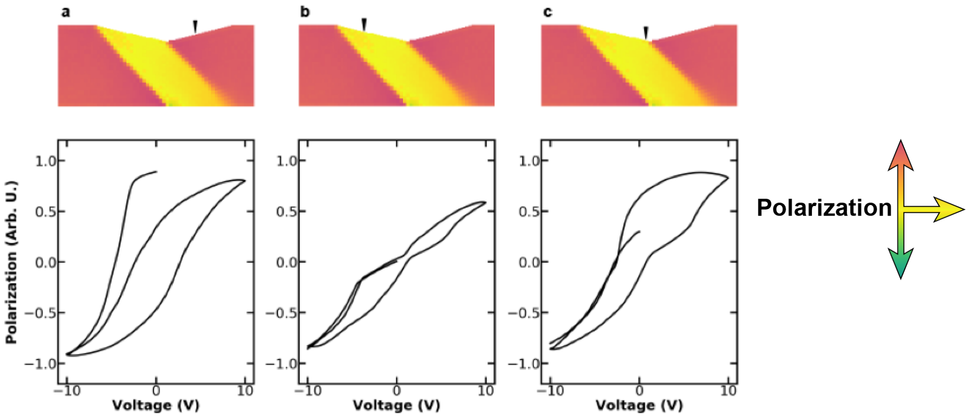

Supplementary Figure 10 | Ferroelectric hysteresis loops extracted from phase-field simulations at various tip positions along the domain wall geometry. Polarization direction in film is indicated on the right. Surface topography is projected from the lattice parameters of the film.

trend that is observed experimentally (Fig. 4). We do note some differences particularly in comparison to the peak boundary. This difference is likely correlated to the difference in how the loop is constructed. In phase-field simulations the loops are calculated from the finite volume average of the polarization in close proximity to the tip. In turn, this is a measure of only the intrinsic response. In piezoresponse measurements we are measuring the change in the piezoresponse during switching which has both long-range and extrinsic contributions. It is likely that these long-range and extrinsic contributions contribute to the hysteresis-like shape observed in the piezoresponse loop which is not observed in the phase field simulation results.

Supplementary References:

1. Gruverman, A., Auciello, O. & Tokumoto, H. Imaging and control of domain structures in ferroelectric thin films via scanning force microscopy. *Annu. Rev. Mater. Sci.* **28,** 101–123 (1998).
2. Jesse, S. & Kalinin, S. V. Band excitation in scanning probe microscopy: sines of change. *J. Phys. D. Appl. Phys.* **44,** 464006-1–33 (2011).

3. Rodriguez, B. J., Callahan, C., Kalinin, S. V. & Proksch, R. Dual-frequency resonance-tracking atomic force microscopy. *Nanotechnology* **18,** 475504-1–5 (2007).

4. Labuda, A. & Proksch, R. Quantitative measurements of electromechanical response with a combined optical beam and interferometric atomic force microscope. *Appl. Phys. Lett.* **106,** 253103-1–4 (2015).

5. Pedregosa, F. *et al.* Scikit-learn: Machine Learning in Python. *J. Mach. Learn. Res.* **12,** 2825–2830 (2012).
