# Supplementary material for: Revealing ferroelectric switching character using deep recurrent neural networks
Source: Nat Commun. 2019 Oct 22;10:4809. doi: 10.1038/s41467-019-12750-0 (PMC6805893; doi:10.1038/s41467-019-12750-0)
Supplement: Supplementary file 2 — Description of Additional Supplementary Files [file 41467_2019_12750_MOESM2_ESM.pdf]

Description of Additional Supplementary Files

File name: Supplementary Movie 1
Description: Raw image series obtained from band excitation piezoresponse force microscopy of PbZr0.2Ti0.2O3 thin films with hierarchical domain structures. Image series of the amplitude, phase, resonance, and quality factor of the cantilever resonance is shown. Bottom figure tracks the progress of switching using a bipolar triangular waveform.

File name: Supplementary Movie 2
Description: Image series showing the activation or output from the piezoresponse autoencoder low-dimensional layer during training. Images are provided when an epoch results in a reduction in the loss. This movie allows for the visualization of the learning process as well as the effect of the sparsity constraint.

File name: Supplementary Movie 3
Description: Image series showing the activation or output from the resonance autoencoder low-dimensional layer during training. Images are provided when an epoch results in a reduction in the loss. This movie allows for the visualization of the learning process as well as the effect of the sparsity constraint.

File name: Supplementary Movie 4
Description: Movie showing the generated piezoresponse curves obtained from the autoencoder as a single activation is changed. The activation changing is represented by the activation map shown in the inset. The colors of the curve match the colors presented in their related activation maps. This movie provides a way to visualize how the activation (as shown in the activation maps) influences the piezoresponse.

File name: Supplementary Movie 5
Description: Movie showing the generated resonance response obtained from the autoencoder as a single activation is changed. The activation changing is represented by the activation map shown in the inset. The colors of the curve match the colors presented in their related activation maps. This movie provides a way to visualize how the activation (as shown in the activation maps) influences the resonance response.

File name: Supplementary Movie 6
Description: Movie showing phase-field simulations of tip induced ferroelectric switching in PbZr0.2Ti0.2O3 thin films when the tip is positioned within a c-like domain near the valley c/a/c/a / a1/a2 domain wall. Movie shows the evolution of the polarization and landau, elastic, electrostatic

and total energy. The left plots indicate the position during switching on a schematic ferroelectric hysteresis loop and bipolar triangular waveform.

File name: Supplementary Movie 7
Description: Movie showing phase-field simulations of tip induced ferroelectric switching in PbZr0.2Ti0.2O3 thin films when the tip is positioned within a-like domain near the peak c/a/c/a / a1/a2 domain wall. Movie shows the evolution of the polarization and landau, elastic, electrostatic and total energy. The left plots indicate the position during switching on a schematic ferroelectric hysteresis loop and bipolar triangular waveform.

File name: Supplementary Movie 8
Description: Movie showing phase-field simulations of tip induced ferroelectric switching in PbZr0.2Ti0.2O3 thin films when the tip is positioned within a-like domain near the valley c/a/c/a / a1/a2 domain wall. Movie shows the evolution of the polarization and landau, elastic, electrostatic and total energy. The left plots indicate the position during switching on a schematic ferroelectric hysteresis loop and bipolar triangular waveform.
